# Vascularized lymph node transfer and lymphovenous anastomosis: temporal microsurgical outcomes in upper versus lower extremity lymphedema^✰^

**DOI:** 10.1016/j.jpra.2026.10.011

**Published:** 2026-10-20

**Authors:** Katrin Seidenstuecker, Séverin R. Wendelspiess, Adriano Fabi, Yvonne Haas, Julia Stoffel, Christoph Kettelhack, Beat P. Müller, Florian S. Halbeisen, Dirk J. Schaefer, Alina Abu-Ghazaleh, Birgit Aufmesser-Freyhardt, Christoph Andree, Benedetta Vasselli, Elisabeth A. Kappos

**Affiliations:** a Department of Plastic, Reconstructive, and Aesthetic Surgery, Sana Kliniken Düsseldorf, Düsseldorf, Germany; b Breast Center, University Hospital Düsseldorf, Düsseldorf, Germany; c Department of Plastic, Reconstructive, Aesthetic and Hand Surgery, University Hospital Basel, Basel, Switzerland; d Faculty of Medicine, University of Basel, Basel, Switzerland; e Department of Surgery, Clarunis - St. Clara Hospital and University Hospital Basel, Basel, Switzerland; f Surgical Outcome Research Center, University Hospital Basel and University of Basel, Basel, Switzerland

**Keywords:** Vascularized lymph node transfer, Lymphovenous anastomosis, Upper extremity, Lower extremity, Chronic lymphedema

## Abstract

**Background:**

Growing evidence supports lymphovenous anastomosis (LVA) and vascularized lymph node transfer (VLNT) in lymphedema. However, little is known about the onset of clinical improvement and temporal profile between upper and lower extremity lymphedema. This study aimed to compare the short- and long-term effectiveness of LVA and VLNT between extremities in patients with secondary lymphedema.

**Methods:**

A prospectively managed database was used to identify patients treated with unilateral microsurgical reconstruction for chronic secondary lymphedema of the upper or lower extremity between January 2016 and December 2023. Patient-specific variables, including circumferential measurements at predefined anatomical landmarks on both affected and unaffected limbs, were recorded preoperatively and at scheduled intervals between three and 24 months postoperatively for comparative analysis.

**Results:**

Of 206 patients with secondary lymphedema, 54.4% were treated for upper and 45.6% for lower extremity lymphedema. Both LVA and VLNT were associated with significant reductions in excess volume. However, substantially greater treatment responses were observed in the upper extremity, regardless of surgical technique. There was a low over all complication rate of 5.3%.

**Conclusion:**

Patients with upper extremity lymphedema achieve greater improvements after LVA and VLNT than those with lower extremity lymphedema. However, these findings remain hypothesis-generating and must be confirmed by other large-scale studies.

## Abbreviations

BMI: Body mass index
CI: Confidence interval
CDT: Complex decongestive therapy
LVA: Lymphovenous anastomosis
*n*: Number
n/a: Not applicable
QoL: Quality of life
PROMs: Patient reported outcome measures
RERR: Relative excess reduction rate
SD: Standard deviation
VLNT: Vascularized lymph node transfer
VLVT: Vascularized lymph vessel transfers

## Introduction

Chronic lymphedema affects approximately 200 million people globally.^1^^,^^2^ Secondary lymphedema, most commonly resulting from malignancies, lymph node dissections, radiotherapy, or infections, is the leading cause of chronic lymphedema in the Western countries.^3^^,^^4^ Patients experience progressive swelling of the affected extremity, which substantially impacts quality of life (QoL) and imposes a significant burden on the healthcare system.^5^^,^^6^ The global gold standard in the treatment of chronic lymphedema of the upper and lower extremities involves conservative therapy including costly and time-consuming complex decongestive therapy (CDT), encompassing both compression therapy and manual lymphatic drainage. While conservative treatment methods are non-curative, reconstructive microsurgical procedures such as lymphovenous anastomosis (LVA) and vascularized lymph node transfer (VLNT) or functional lymphatic reconstruction with vascularized lymph vessel transfers (VLVT) have gained significance, as they address the underlying pathomechanism by reconstructing lymphatic pathways.^7^, ^8^, ^9^, ^10^, ^11^, ^12^ LVA is a minimally invasive technique, in which microsurgical anastomoses are created between one or more lymphatic vessels and adjacent low pressure veins, allowing lymphatic fluid to bypass obstructed pathways and improving drainage in the affected area.^13^, ^14^, ^15^ In contrast, VLNT involves harvesting healthy lymph nodes from an unaffected body part and transplanting them into the lymphedematous region with microsurgical vascular anastomosis. The transplanted lymph nodes are believed to restore the lymphatic drainage through gradual lymphangiogenesis.^16^, ^17^, ^18^

Both techniques are considered safe and effective, with promising clinical outcomes and improvements in patients’ QoL.^11^^,^^19^, ^20^, ^21^, ^22^ Nevertheless, there is a scarcity of studies comparing the potential of lymphatic reconstruction in the upper versus lower extremities. Therefore, the aim of this study was to compare the short- and long-term effectiveness of LVA and VLNT in patients with secondary lymphedema of the upper or lower extremity.

## Material and methods

This study was conducted in accordance with the World Medical Association Declaration of Helsinki^23^ and was approved by the regional ethics committee.

### Study design and outcomes

This retrospective cohort study screened a prospectively maintained database for patients who underwent unilateral LVA or VLNT for chronic secondary lymphedema of the upper or lower extremity between January 2016 and December 2023. Written informed consent for inclusion in the study was obtained from all patients before participation. Patients were eligible if they had at least one documented postoperative assessment with a measured RERR. Follow-up visits were scheduled at 3, 6, 12, and 24 months. Accordingly, "minimum follow-up" refers to the availability of at least one postoperative measurement rather than completion of the full 24-month schedule. Patients with primary lymphedema or an undocumented etiology, bilateral lymphedema, bilateral interventions, those with additional liposuction and/or without written informed consent were excluded. Etiology was classified from the documented clinical history, i.e. a preceding oncological, surgical, radiotherapeutic or infectious trigger. Patients in whom no trigger could clearly be identified were not classified as secondary and were excluded. Restricting the cohort to secondary lymphedema removes the principal source of pathophysiological heterogeneity from the between-extremity comparison. The circumferences of both affected and unaffected limbs were measured at specific reference points, including the maximum width of the hand or foot, wrist or ankle and additional landmarks at 10 cm intervals extending proximally up to 50–60 cm from the wrist or ankle. All measurements were performed by specially trained plastic surgeons following the standardized protocols preoperatively and at the regular follow-up visits at 3, 6, 12 and 24 months. Patients' characteristics, such as demographics and clinical parameters, including age, Body Mass Index (BMI) and postoperative complications graded according to the Clavien-Dindo classification,^24^ were extracted from medical records. The primary outcome was the comparison of the effectiveness of LVA and VLNT between the upper and lower extremity over time measured by the relative excess reduction rates (RERR). Because procedure selection was clinically indicated rather than randomized, the analysis was conducted as a two-group longitudinal comparison and is not intended to establish superiority of one technique over the other.

### Surgical procedure and postoperative management

All patients underwent a standardized preoperative work-up comprising clinical history, clinical staging, standardized circumferential measurement of both limbs and, where clinically indicated and available, preoperative lymphatic imaging. Procedure selection (LVA vs. VLNT) was individualized based on lymphedema stage/severity and the quality of functional lymphatic vessels: LVA was preferred in less advanced disease with suitable collectors, whereas VLNT was used for more advanced disease and/or for poor lymphatic vessel quality. All procedures were performed by the same two surgeons, who share similar microsurgical training background, resulting in comparability of technique. Per case, 2–4 LVAs were created according to the intraoperative availability of suitable lymphatic vessels and recipient veins. VLNT was performed in an analogous standardized manner; when abdominal lymph nodes (omental and mesenteric) were harvested, a two-team approach with visceral surgeons was used. Details of the surgical procedure have been published previously.^21^ Postoperative management included standardized continuation of compression therapy and physiotherapy for all participants. In accordance with our institutional protocol, patients were instructed to wear compression garments continuously (24 h per day) during the first six postoperative weeks. After this initial period, nighttime compression could be reduced, while daytime compression (approximately 12 h daily, seven days per week) was maintained. Beginning six months postoperatively, patients with upper extremity lymphedema were gradually weaned from compression therapy, whereas patients with lower extremity lymphedema were advised to continue lifelong compression therapy.

### Relative excess reduction rate

To quantify changes in excess volume compared to the unaffected limb, RERR were used and calculated at each postoperative follow-up using the following formula^22^

$$RERR=\left(1-\frac{⌀\text{Circumference}\;\text{month}\;x\;\left(\text{affected}\;\text{limb}\right)-\;⌀\text{Circumference}\;\text{month}\;x\;\left(\text{unaffected}\;\text{limb}\right)}{\left|⌀\text{Circumference}\;\text{preop}.\;\left(\text{affected}\;\text{limb}\right)-\;⌀\text{Circumference}\;\text{preop}.\;\left(\text{unaffected}\;\text{limb}\right)\right|}\right)*100*\left(-1\right)$$

For each limb, the circumferences measured at the predefined landmarks were aggregated as an arithmetic mean (⌀), and the RERR was calculated from the mean-circumference difference between the affected and unaffected limb, normalized to the preoperative excess. The unaffected contralateral limb served as an internal reference for each patient. By this definition, a negative RERR reflects a reduction of the excess circumference relative to baseline (i.e., clinical improvement), a value of zero indicates no change, and a positive value indicates an increase in excess circumference. Here, ⌀ circumference denotes the arithmetic mean of the circumference measurements obtained at the predefined anatomical landmarks (at 10-cm intervals) along the limb. Circumference-based assessment is a low-cost and reproducible standard for quantifying lymphedema and was the routinely available measurement modality at our institution. Perometry, bioimpedance spectroscopy, volumetric imaging and water-displacement volumetry were not routinely available. For brevity, reductions in the circumference-derived excess are hereafter referred to as reductions in excess volume. This terminology reflects a circumference-based surrogate rather than a direct volumetric measurement.

### Statistical analysis

Descriptive statistics were used to summarize patient characteristics. Results were presented as frequencies with percentages for categorical variables and means or medians with standard deviation or interquartile ranges (IQR) for continuous variables. Categorical variables were compared between groups using Fisher’s exact test, and continuous variables were analyzed using the Wilcoxon rank-sum test.

To assess changes in RERR over time and between intervention groups, we used linear mixed-effects models with a random intercept for each patient. Time was modeled as a categorical variable (3, 6, 12, 24 months; 3 months as reference), and fixed effects included intervention (LVA/VLNT) and extremity (upper/lower) together with their two- and three-way interactions with time. Baseline excess is intrinsic to the RERR outcome and was therefore not added as a separate covariate. Estimated marginal means with 95% confidence intervals were derived from the fitted models; p-values for the time, group and interaction terms are reported. Full model coefficients are provided in Supplementary Tables S1–S3. In addition, the proportion of patients with any reduction of excess volume (RERR < 0) was compared between LVA and VLNT at each timepoint using Fisher's exact test. p-values ≤ 0.05 were considered statistically significant. All statistical analyses were performed using the R statistical software (Version 4.3.2, The R Foundation for Statistical Computing, Vienna, Austria).^25^

## Results

Of 265 screened patients, 59 were excluded because of primary (n = 57) or undocumented (n = 2) etiology, leaving 206 patients with secondary extremity lymphedema for analysis. Of these, 192 (93.2%) were female and 14 (6.8%) were male. Overall, 112 (54.4%) patients suffered from chronic lymphedema of the upper extremity and 94 (45.6%) of the lower extremity. There was no statistical difference in age between the extremity groups (upper 54.3 ± 10.0 versus lower 52.2 ± 11.2 years, p = 0.147). However, patients with upper extremity lymphedema had a higher body mass index (27.1 ± 4.6 versus 24.9 ± 4.4 kg/m2, p < 0.001; Table 1). In the lower extremity, patients undergoing LVA had a lower BMI than those treated with VLNT (24.4 ± 4.3 versus 26.7 ± 4.6 kg/m2, p = 0.017; Table 2). Most patients suffered from stage II lymphedema, with no significant difference in staging between upper and lower extremities (p = 0.849). LVA was the most common surgical technique in the lower extremity (78.7%), whereas VLNT predominated in the upper extremity (63.4%; p < 0.001, Table 1).

**Table 1:** Patients' characteristics in the upper versus lower extremity groups.

Characteristic	Lower extremity (n = 94)	Upper extremity (n = 112)	p-value*
Age, mean years (±SD)	52.2 (± 11.2)	54.3 (± 10.0)	0.147
BMI, mean kg/m2 (±SD)	24.9 (± 4.4)	27.1 (± 4.6)	<0.001
Sex			0.054
Male	10 (10.6)	4 (3.6)	
Female	84 (89.4)	108 (96.4)	
Lymphedema stage			0.849
Stage I	1 (1.1)	3 (2.7)	
Stage II	91 (96.8)	105 (93.8)	
Stage III	2 (2.1)	2 (1.8)	
N/A	0 (0)	2 (1.8)	
Surgical technique			<0.001
LVA	74 (78.7)	41 (36.6)	
VLNT	20 (21.3)	71 (63.4)	

Description: Values are reported as absolute count (%) unless reported otherwise. BMI Body mass index; CI Confidence interval; LVA Lymphovenous anastomosis; N/A Not applicable; n Number; RERR Relative excess reduction rate; SD Standard deviation; VLNT Vascularized lymph node transfer. * Wilcoxon rank-sum test for continuous and Fisher's exact test for categorical variables.

**Table 2:** Differences between LVA and VLNT within the lower and the upper extremity.

Characteristic	Lower extremity		p-value*	Upper extremity		p-value*
	LVA (n = 74)	VLNT (n = 20)		LVA (n = 41)	VLNT (n = 71)	
Sex			0.683			0.016
Male	9 (12.2)	1 (5.0)		4 (9.8)	0 (0)	
Female	65 (87.8)	19 (95.0)		37 (90.2)	71 (100)	
Age, mean years (±SD)	52.2 (± 10.9)	52.3 (± 12.5)	0.831	54.1 (± 11.5)	54.5 (± 9.0)	0.876
BMI, mean kg/m2 (±SD)	24.4 (± 4.3)	26.7 (± 4.6)	0.017	26.8 (± 5.4)	27.3 (± 4.0)	0.642
Lymphedema stage			0.507			0.028
Stage I	1 (1.4)	0 (0)		3 (7.3)	0 (0)	
Stage II	72 (97.3)	19 (95.0)		38 (92.7)	67 (94.4)	
Stage III	1 (1.4)	1 (5.0)		0 (0)	2 (2.8)	
N/A	0 (0)	0 (0)		0 (0)	2 (2.8)	

Description: Values are reported as absolute count (%) unless reported otherwise. BMI Body mass index; CI Confidence interval; LVA Lymphovenous anastomosis; N/A Not applicable; n Number; RERR Relative excess reduction rate; SD Standard deviation; VLNT Vascularized lymph node transfer. * Wilcoxon rank-sum test for continuous and Fisher's exact test for categorical variables.

### Overall effectiveness of LVA and VLNT in the lower versus upper extremity

Both LVA and VLNT were associated with a reduction of excess volume at most timepoints in the upper and lower extremities (Fig. 1, Supplementary Table S1). In the combined model, neither the main effect of procedure (VLNT versus LVA, p = 0.498) nor the procedure-by-time interaction (p = 0.229) reached statistical significance, whereas the extremity-by-time interaction did (p = 0.022), indicating that the temporal course rather than the overall magnitude of the response differed between extremities. The three-way interaction between time, procedure and extremity was not significant (p = 0.912). At the 24-month visit, 33 of 40 assessed patients (82.5%) had a RERR < 0, indicating a reduction of excess volume, whereas 7 (17.5%) had a RERR ≥ 0. The number of patients assessed at each follow-up and the corresponding proportions of improvement are shown in Table 4. Numbers were small at the 3-month (n = 49) and 24-month (n = 40) visits, and all estimates for these timepoints must be interpreted accordingly.

**Fig. 1:**
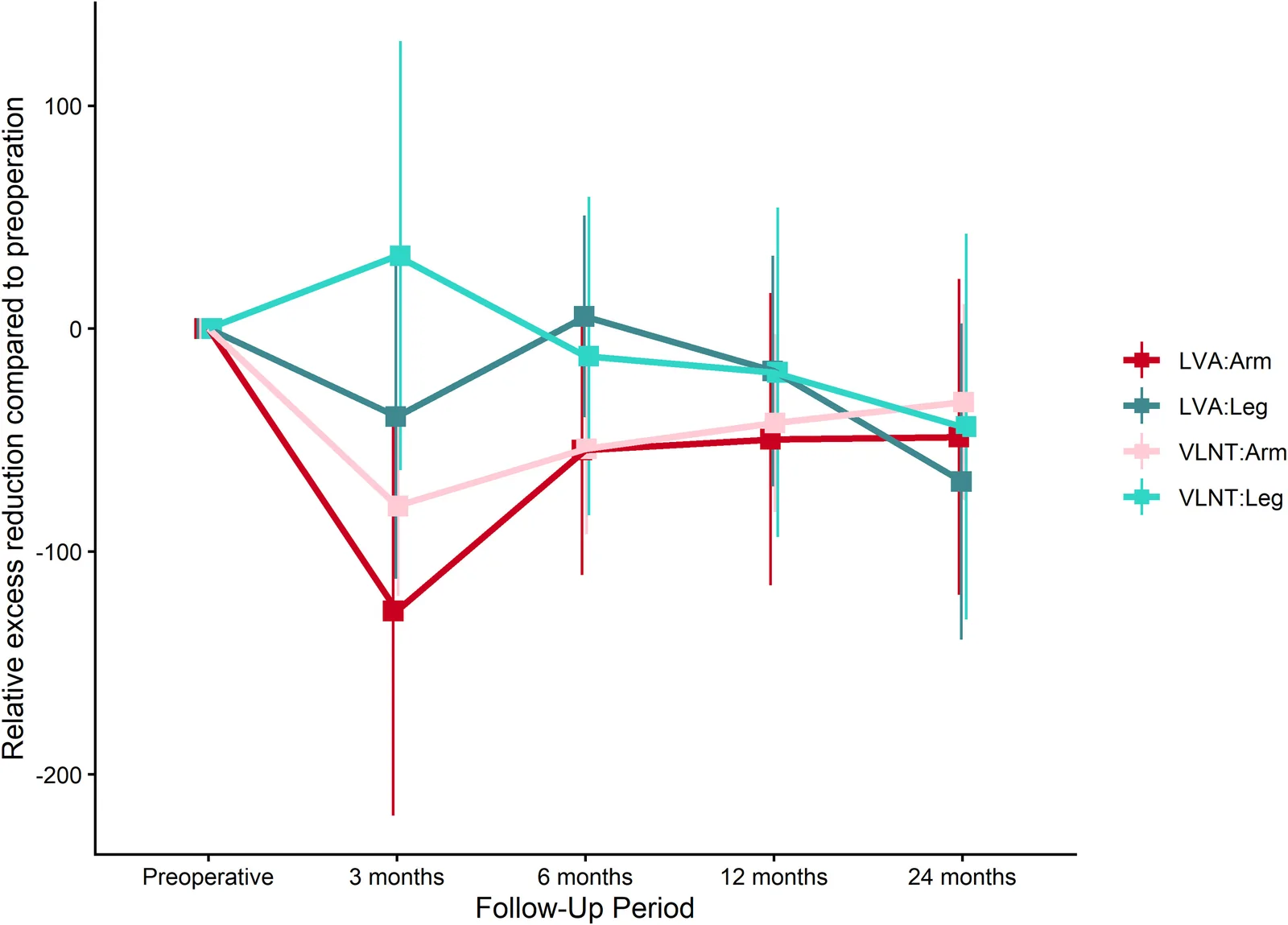
Relative excess reduction rate (%) over time for LVA and VLNT in the upper and lower extremity Description: Estimated marginal means with 95% confidence intervals from a linear mixed-effects model with a random intercept per patient. More negative values indicate a greater reduction of excess volume relative to the preoperative baseline. LVA Lymphovenous anastomosis; VLNT Vascularized lymph node transfer.

### LVA: Effectiveness in the lower versus upper extremity

LVA was performed in 115 (55.8%) patients, including 74 (64.3%) with lower and 41 (35.7%) with upper extremity lymphedema. The mean number of anastomoses was higher in the lower extremity (2.84 ± 0.96 versus 2.44 ± 0.73, p = 0.027; Table 3). Follow-up was sparse in both groups: 11 of 41 upper-extremity (26.8%) and 18 of 74 lower-extremity (24.3%) LVA patients were assessed at 12 months, and 8 (19.5%) and 6 (8.1%), respectively, at 24 months. Within the first three months, the estimated RERR was more pronounced in the upper extremity (−127.2%, 95% CI −235.2 to −19.3) than in the lower extremity (−41.2%, 95% CI −128.4 to +46.0). These estimates rest on three upper- and seven lower-extremity measurements. The early upper-extremity effect attenuated thereafter, reaching −50.4% (95% CI −139.5 to +38.8) at 24 months. In the lower extremity, the estimate fluctuated around zero at six months (+3.7%, 95% CI −58.1 to +65.6) and became more negative towards 24 months (−53.2%, 95% CI −137.6 to +31.2). Neither the extremity main effect (p = 0.393) nor the extremity-by-time interaction (p = 0.449) was statistically significant in the LVA-only model, and all confidence intervals except the 3-month upper-extremity estimate included zero (Table 5, Fig. 2, Supplementary Table S2).

**Table 3:** LVA patient characteristics in the lower versus upper extremity groups.

LVA	Lower extremity (n = 74)	Upper extremity (n = 41)	p-value*
Age, mean years (±SD)	52.2 (± 10.9)	54.1 (± 11.5)	0.487
BMI, mean kg/m2 (±SD)	24.4 (± 4.3)	26.8 (± 5.4)	0.012
Anastomoses, n (±SD)	2.84 (± 0.96)	2.44 (± 0.73)	0.027
Lymphedema stage			0.180
Stage I	1 (1.4)	3 (7.3)	
Stage II	72 (97.3)	38 (92.7)	
Stage III	1 (1.4)	0 (0)	

Description: Values are reported as absolute count (%) unless reported otherwise. BMI Body mass index; CI Confidence interval; LVA Lymphovenous anastomosis; n Number; SD Standard deviation; * Wilcoxon rank-sum test for continuous and Fisher's exact test for categorical variables.

**Table 4:** Number of patients assessed and proportion with RERR improvement at each follow-up, by procedure and extremity.

Time point	LVA, N	LVA improved, n (%)	VLNT, N	VLNT improved, n (%)	p-value*
Both extremities					
3 months	10	8 (80.0)	39	35 (89.7)	0.588
6 months	63	45 (71.4)	67	50 (74.6)	0.697
12 months	29	22 (75.9)	50	37 (74.0)	1.00
24 months	14	13 (92.3)	26	20 (76.9)	0.387
Upper extremity					
3 months	3	3 (100)	35	32 (91.4)	1.00
6 months	25	21 (84.0)	52	43 (82.7)	1.00
12 months	11	9 (81.8)	38	31 (81.6)	1.00
24 months	8	7 (87.5)	21	16 (76.2)	0.647
Lower extremity					
3 months	7	5 (71.4)	4	3 (75.0)	1.00
6 months	38	24 (63.2)	15	7 (46.7)	0.357
12 months	18	13 (72.2)	12	6 (50.0)	0.266
24 months	6	6 (100)	5	4 (80.0)	0.455

Improvement was defined as RERR < 0 (reduction of excess circumference relative to baseline). N denotes the number of patients with an available RERR measurement at the respective timepoint. * Fisher's exact test comparing the proportion of improved patients between LVA and VLNT.

**Table 5:** Relative excess reduction rates [%] by extremity for LVA over time.

Location	Time	Estimate	Lower 95% CI	Upper 95% CI
Upper extremity				
	3 months	−127.2	−235.2	−19.3
	6 months	−54.0	−130.7	+22.8
	12 months	−51.1	−135.6	+33.4
	24 months	−50.4	−139.5	+38.8
Lower extremity				
	3 months	−41.2	−128.4	+46.0
	6 months	+3.7	−58.1	+65.6
	12 months	−18.8	−85.9	+48.3
	24 months	−53.2	−137.6	+31.2

Estimated marginal means with 95% confidence intervals from a linear mixed-effects model with a random intercept per patient, restricted to patients treated with LVA. More negative values indicate greater reduction of excess volume. Extremity main effect p = 0.393; extremity-by-time interaction p = 0.449. CI Confidence interval.

**Fig. 2:**
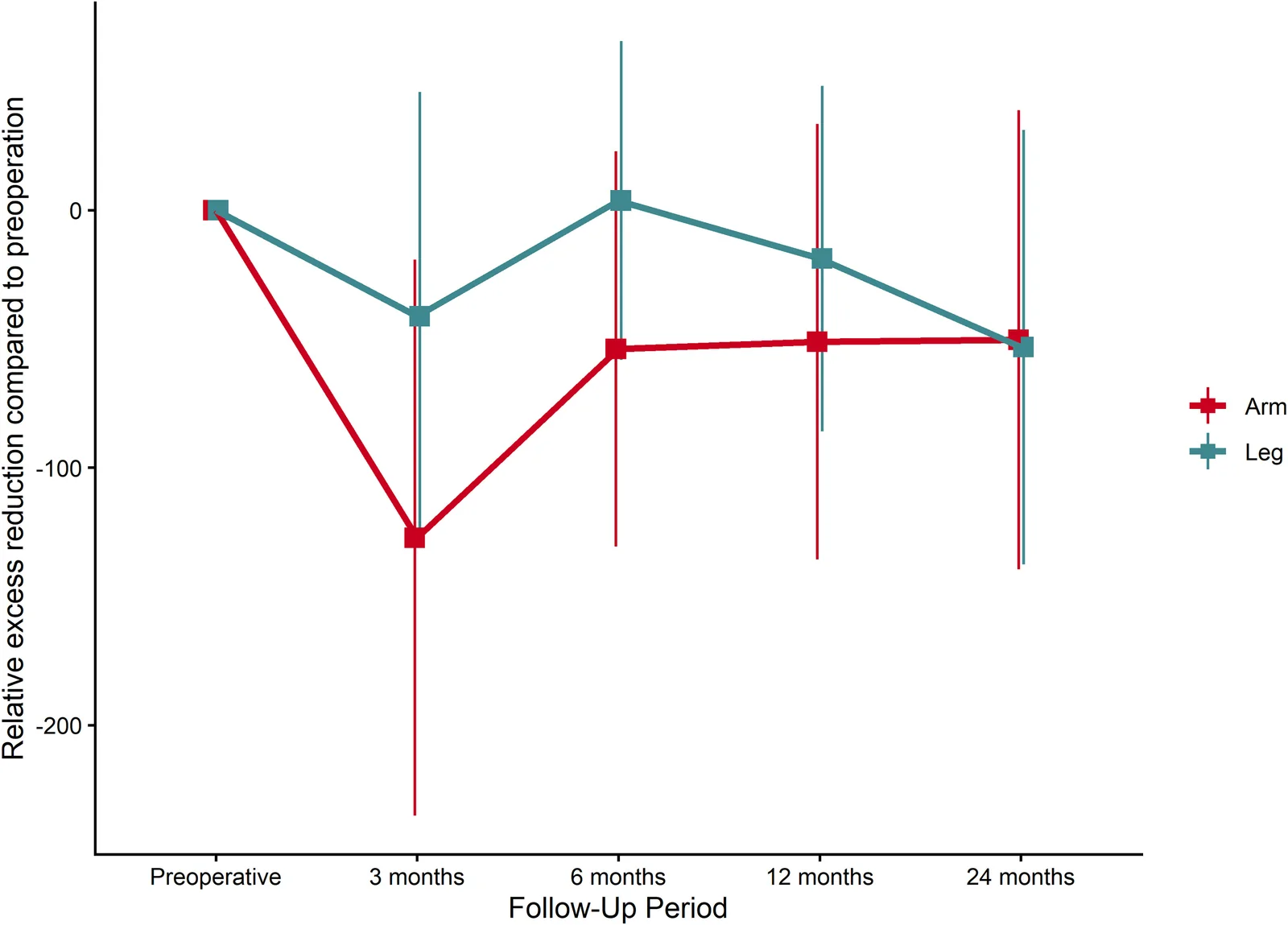
Relative excess reduction rate (%) by extremity over time for LVA Description: Estimated marginal means with 95% confidence intervals from a linear mixed-effects model restricted to patients treated with LVA (n = 115). LVA Lymphovenous anastomosis.

### VLNT: Effectiveness in the lower versus upper extremity

VLNT was performed in 91 (44.2%) patients, including 71 (78.0%) in the upper and 20 (22.0%) in the lower extremity. In the upper extremity, lymph nodes were almost exclusively harvested from the groin (68 of 71, 95.8%), whereas in the lower extremity the thoracic (13 of 20, 65.0%) and abdominal (mesenteric 6 of 20, 30.0%) donor sites predominated (p < 0.001; Table 6). Follow-up rates were higher than after LVA: 38 of 71 upper-extremity (53.5%) and 12 of 20 lower-extremity (60.0%) patients were assessed at 12 months, and 21 (29.6%) and 5 (25%), respectively, at 24 months. In the upper extremity, all estimated RERRs differed significantly from zero: −83.8% (95% CI −108.4 to −59.3) at three months, −51.1% (−72.4 to −29.9) at six months, −43.8% (−67.6 to −20.0) at 12 months and −32.7% (−62.5 to −2.9) at 24 months, indicating a sustained but progressively attenuating effect. In the lower extremity, the estimates moved in the opposite direction over time, from +12.7% (−56.7 to +82.1) at three months to −38.0% (−98.7 to +22.8) at 24 months, but none reached statistical significance. The extremity main effect was significant in the VLNT-only model (p = 0.045), reflecting the more favourable early estimates in the upper extremity, whereas the extremity-by-time interaction was not (p = 0.178) (Table 7, Fig. 3, Supplementary Table S3).

**Table 6:** VLNT patient characteristics in the lower versus upper extremity groups.

VLNT	Lower extremity (n = 20)	Upper extremity (n = 71)	p-value*
Age, mean years (±SD)	52.3 (± 12.5)	54.5 (± 9.0)	0.411
BMI, mean kg/m2 (±SD)	26.7 (± 4.6)	27.3 (± 4.0)	0.325
Lymphedema stage			0.539
Stage II	19 (95.0)	67 (94.4)	
Stage III	1 (5.0)	2 (2.8)	
N/A	0 (0)	2 (2.8)	
Donor sites			<0.001
Groin	1 (5.0)	68 (95.8)	
Lateral thoracic wall	13 (65.0)	0 (0)	
Mesenterium	6 (30.0)	1 (1.4)	
Omentum	0 (0)	2 (2.8)	
Recipient sites			<0.001
Groin	8	–	
Lower leg	12	–	
Axilla	–	65 (91.5)	
Axilla + forearm	–	1 (1.4)	
Forearm	–	5 (7.0)	

Values are reported as absolute count (%) unless reported otherwise. BMI Body mass index; CI Confidence interval; N/A Not applicable; n Number; SD Standard deviation; VLNT Vascularized lymph node transfer. * Wilcoxon rank-sum test for continuous and Fisher's exact test for categorical variables.

**Table 7:** Relative excess reduction rates [%] by extremity for VLNT over time.

Location	Time	Estimate	Lower 95% CI	Upper 95% CI
Upper extremity				
	3 months	−83.8	−108.4	−59.3
	6 months	−51.1	−72.4	−29.9
	12 months	−43.8	−67.6	−20.0
	24 months	−32.7	−62.5	−2.9
Lower extremity				
	3 months	+12.7	−56.7	+82.1
	6 months	−9.8	−49.5	+29.8
	12 months	−12.2	−55.4	+31.0
	24 months	−38.0	−98.7	+22.8

Estimated marginal means with 95% confidence intervals from a linear mixed-effects model with a random intercept per patient, restricted to patients treated with VLNT. More negative values indicate greater reduction of excess volume. Extremity main effect p = 0.045; extremity-by-time interaction p = 0.178. CI Confidence interval.

**Fig. 3:**
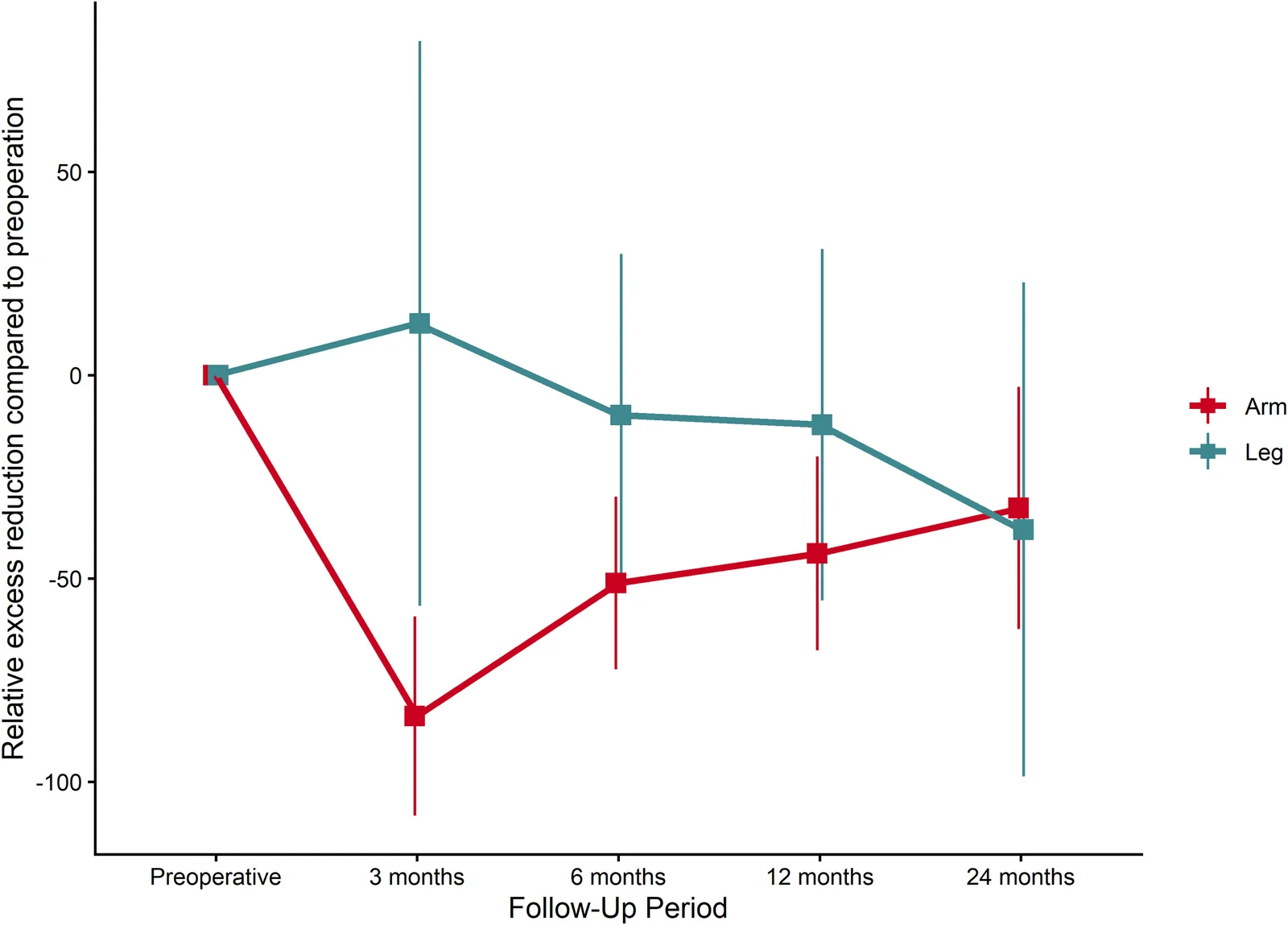
Relative excess reduction rate (%) by extremity over time for VLNT Description: Estimated marginal means with 95% confidence intervals from a linear mixed-effects model restricted to patients treated with VLNT (n = 91). VLNT Vascularized lymph node transfer.

### Complications

A total of 11 complications were observed in 206 patients (5.3%), all of which occurred in the VLNT group (11 of 91 VLNT patients, 12.1%). Nine complications (9 of 71, 12.7%) occurred in the upper extremity and two (2 of 20, 10.0%) in the lower extremity, the latter both being minor wound healing disorders (Table 8). The majority (n = 9, 81.8%) of these complications were classified as Clavien-Dindo grade II or lower and therefore did not require surgical revision. Recipient-site complications accounted for seven of the 11 events and comprised two flap-related events requiring surgical revision (one total and one partial flap loss), both in the upper extremity. There were five minor wound healing complications (four wound healing disorders and one infection). Two hematomas and two seromas, both graded as Clavien-Dindo grade II and observed in the upper extremity, could not be localized to donor or recipient site based on the available records. No complications occurred in patients undergoing LVA.

**Table 8:** Type of complication by intervention and extremity.

Clavien-Dindo	Type of complication	Lower extremity		Upper extremity		p-value*
		LVA (n = 74)	VLNT (n = 20)	LVA (n = 41)	VLNT (n = 71)	
II	Wound healing disorder	0 (0)	2 (10.0)	0 (0)	2 (2.8)	
II	Hematoma	0 (0)	0 (0)	0 (0)	2 (2.8)	
II	Seroma	0 (0)	0 (0)	0 (0)	2 (2.8)	
II	Wound infection	0 (0)	0 (0)	0 (0)	1 (1.4)	
IIIb	Partial flap loss	0 (0)	0 (0)	0 (0)	1 (1.4)	
IIIb	Total flap loss	0 (0)	0 (0)	0 (0)	1 (1.4)	
	Any complication	0 (0)	2 (10.0)	0 (0)	9 (12.7)	1.00

Values are reported as absolute count (% of the respective group). * Fisher's exact test comparing the complication rate after VLNT between the lower and the upper extremity. LVA Lymphovenous anastomosis; VLNT Vascularized lymph node transfer.

## Discussion

Our findings indicate that both LVA and VLNT are associated with significant reductions of excess limb volume in secondary lymphedema of either extremity. However, patients with upper extremity lymphedema demonstrated greater treatment responses than those with lower extremity lymphedema, regardless of surgical technique.

### Effectiveness of LVA and VLNT in the upper versus the lower extremity

In the present cohort, restricted to secondary lymphedema, LVA showed its largest estimated reduction of excess volume in the upper extremity within the first three months. Importantly, the numbers of anastomoses was slightly higher in the lower than in the upper extremity (2.84 ± 0.96 vs. 2.44 ± 0.73, p = 0.027). Consequently, the real effect may be even larger, when adjusting for the number of anastomoses performed. Interestingly, the effectiveness plateaued after 6 months, suggesting a potential stagnation of clinical improvements. Published data suggest that the effect of LVA may attenuate during the first postoperative year in upper-extremity lymphedema. Importantly, Winters et al. reported an initial volume reduction of 25.5% within six months, decreasing to 8.9% over the subsequent six months, with anastomotic patency preserved in 56% of cases at 12 months.^26^ Similarly, Maegawa et al. reported a 12-month patency rate of 75% in peripheral lymphedema.^27^ These studies indicate that loss of LVA patency may potentially explain the plateau observed in our cohort.

In contrast, VLNT showed a distinct descriptive temporal pattern between extremities. In the upper extremity, RERR was significantly reduced at all timepoints but gradually attenuated from −83.8% at three months to −32.7% at 24 months. In contrast, lower-extremity estimates became progressively more negative over time, reaching −38.0% at 24 months. While the extremity main effect was significant (p = 0.045), the extremity-by-time interaction was not (p = 0.178), and the three-way multivariable adjustment between time, procedure, and extremity was likewise non-significant (p = 0.912). The pronounced early effect in the upper extremity may reflect axillary scar release, which may immediately improve lymphatic drainage.^28^ Maruccia et al. similarly reported greater volume reduction when VLNT was combined with axillary scar release than with VLNT alone in breast cancer-related lymphedema.^29^ Similarly, the two extremity groups differed in recipient sites, with groin-to-axilla transfer in virtually all upper-extremity cases versus thoracic or abdominal donor sites combined with both proximal and distal recipient sites in the lower extremity. Moreover, intra-abdominal scar-tissue release could not performed in the lower extremity, which may have influenced the temporal course of postoperative changes.^28^ The loss of effectiveness may also be influenced by postoperative compression adherence. Although evidence on continued conservative therapy after lymphatic surgery remains limited, Hahn et al. reported greater volume reduction with continued compression following surgery for chronic lower-extremity lymphedema.^30^ According to our institutional protocol, compression therapy is gradually tapered after six months in the upper extremity, whereas it is continued indefinitely in the lower extremity. Despite this less intensive long-term compression regimen, outcomes remained more favorable in the upper extremity, suggesting that the observed effectiveness in this group may even be underestimated. This finding further supports the possibility that the differing temporal profiles are related to lower-limb–specific physiological conditions, including greater limb volume and mass, gravity-dependent hydrostatic loading with higher venous pressures, and an increased tendency toward dependent fluid stasis compared with the upper extremity.^31^

### Complication rates

An overall complication rate of 5.3% (n = 11 of 206) was observed, none of which was attributed to the LVA group, underscoring its safety and minimally invasive nature.^11^^,^^20^ The slightly higher complication rate associated with VLNT may reflect its greater surgical complexity and longer operation duration.^18^^,^^21^^,^^32^ Considering the total of 71 VLNT procedures performed on the upper extremity and 20 on the lower extremity, complication rates appear comparable, with nine complications reported in the upper (12.7%) and two in the lower extremity (10.0%); with two events in the lower extremity group, this comparison is statistically uninformative (Fisher’s exact test, p = 1.00). Several factors may contribute to complications at axillary recipient site. Firstly, the upper extremity, particularly the axillary region, undergoes greater mechanical stress due to a wider range of motion, potentially compromising vascular anastomoses.^33^ Secondly, surgical complexity is often higher in the axillary region due to complex anatomy and extensive scar tissue resulting from postoperative radiotherapy in patients with breast cancer-related lymphedema.^34^

### Study strengths and limitations

This study offers novel insights into the comparative effectiveness of lymphatic reconstruction in the upper versus the lower extremity. To the best of our knowledge, this is the first study to compare both surgical techniques across upper and lower extremity lymphedema in a sizeable cohort with a follow-up of up to 24 months. Restricting the analysis to secondary lymphedema removes the most important source of pathophysiological heterogeneity between the extremity groups, but several limitations remain. First, the two groups still differ in the distribution of procedures (LVA predominating in the lower and VLNT in the upper extremity) and, within VLNT, in donor and recipient sites; residual confounding by indication therefore cannot be excluded, and the comparison should not be read as a head-to-head comparison of two identical operations. Second, the number of patients assessed at the individual timepoints was small, particularly at three months (n = 49) and at 24 months (n = 40), so that estimates at these visits are imprecise and the 3-month LVA estimates in particular rest on single-digit numbers per group. Third, attrition was substantial and probably non-random: only 25.2% of LVA patients were assessed at 12 months, compared with 54.9% after VLNT. Patients with marked and rapid improvement may have been less likely to return, whereas VLNT involves an additional surgical donor site requiring postoperative surveillance, which may have increased follow-up attendance in that group. Because dropout was probably related to outcome, the long-term trajectory estimates, particularly for LVA, may be biased. No formal comparison of baseline characteristics between patients with and without long-term follow-up and no sensitivity analysis for informative dropout were performed. Fourth, postoperative compression protocols differed between extremities: upper-extremity patients were gradually weaned from compression after six months, whereas lower-extremity patients were advised lifelong compression. As compression independently reduces limb volume, this time-varying co-intervention is a potential source of bias and may itself contribute to the observed attenuation of the upper-extremity response and to the more sustained lower-extremity response, independent of surgical technique. Fifth, outcomes were derived from circumference measurements rather than from validated volumetric methods such as water displacement, perometry or bioimpedance spectroscopy; averaged circumferences may not fully capture segmental changes of the limb. Sixth, PROMs and quality-of-life data were not collected in a standardized manner, although these would be paramount to evaluate surgical success from the patient's perspective. Finally, this was an observational, non-randomized retrospective cohort study. Taken together, these limitations mean that the present findings should be interpreted as a descriptive two-group longitudinal analysis and as hypothesis-generating, rather than as evidence of the superiority of one technique over the other or of definitive long-term efficacy.

## Conclusion

Lymphatic reconstruction achieves greater effectiveness in the upper extremity than the lower extremity for secondary lymphedema, regardless of surgical technique. These patterns may reflect anatomical, physiological, technical, and compression-related differences, but cannot be disentangled within this non-randomized cohort. Prospective, real-world studies with standardized compression protocols, volumetric measurements, and patient-reported outcomes are required to further validate these preliminary findings.

## Declarations

### Ethics approval and consent to participate

This study was conducted in accordance with the principles of the Declaration of Helsinki and was granted ethics approval by the local ethics committee “Ethikkommission Nordwest und Zentralschweiz EKNZ” (BASEC-ID: 2020–01307). All participants gave informed consent before inclusion in this study.

### Availability of data and materials

The data that support the findings of this study are not publicly available pursuant to the ethics committee approval by the “Ethikkommission Nordwest und Zentralschweiz EKNZ” in Switzerland. The data can be obtained from the corresponding author upon reasonable request.

### Statement on AI-assisted technologies in the writing process

During the revision of this work, the authors used Claude (Anthropic) to improve readability. After using this tool, the authors reviewed and edited the content as needed and take full responsibility for the content of the publication.

### Funding

This study was not funded.

## Conflict of interest

None of the contributing authors have any conflicts of interests or financial support to disclose.
