# Supplementary material for: Vascularized lymph node transfer and lymphovenous anastomosis: temporal microsurgical outcomes in upper versus lower extremity lymphedema
Source: JPRAS Open. 2026 Oct 20;53:128–38. doi: 10.1016/j.jpra.2026.10.011 (PMC13761365; doi:10.1016/j.jpra.2026.10.011)
Supplement: Supplementary file 1 [file mmc1.docx]

STROBE Statement—checklist of items that should be included in reports of observational studies

|  | Item No. | Recommendation | Page  No. | Relevant text from manuscript |
| --- | --- | --- | --- | --- |
| **Title and abstract** | 1 | (*a*) Indicate the study’s design with a commonly used term in the title or the abstract | 2,5-6 | The design is stated in the Methods: “This retrospective cohort study screened a prospectively managed database …”. The Abstract Methods describe the data source and the comparative, longitudinal set-up: “A prospectively managed database was used to identify patients treated with unilateral microsurgical reconstruction …”. |
|  |  | (*b*) Provide in the abstract an informative and balanced summary of what was done and what was found | 2 | Conclusion. Findings are reported without superiority claims: “Both LVA and VLNT were associated with excess-volume reduction in both extremities, with comparable estimates at 24 months.” |
| Introduction | | | |  |
| Background/rationale | 2 | Explain the scientific background and rationale for the investigation being reported | 4 | Background and evidence gap stated at the end of the Introduction: “Nevertheless, there is a scarcity of studies comparing the potential of lymphatic reconstruction in the upper versus lower extremities.” |
| Objectives | 3 | State specific objectives, including any prespecified hypotheses | 4 | Objective stated at the end of the Introduction: “Therefore, the aim of this study was to compare the short- and long-term effectiveness of LVA and VLNT in upper- and lower extremities.” No formal statistical hypothesis was prespecified; the analysis is descriptive comparative (see item 4). |
| Methods | | | |  |
| Study design | 4 | Present key elements of study design early in the paper | 5-6 | Section 2.1: “This retrospective cohort study screened a prospectively managed database for patients who underwent unilateral LVA or VLNT …”. The comparative framing is defined explicitly: “Because procedure selection was clinically indicated rather than randomized, the analysis was conducted as a two-group longitudinal comparison and is not intended to establish superiority of one technique over the other.” |
| Setting | 5 | Describe the setting, locations, and relevant dates, including periods of recruitment, exposure, follow-up, and data collection | 5 | Section 2.1: surgery period “between January 2016 and December 2023”; “Follow-up visits were scheduled at 3, 6, 12, and 24 months.” All procedures were performed by the same two surgeons at the participating departments (Section 2.2). |
| Participants | 6 | (*a*) *Cohort study*—Give the eligibility criteria, and the sources and methods of selection of participants. Describe methods of follow-up  *Case-control study*—Give the eligibility criteria, and the sources and methods of case ascertainment and control selection. Give the rationale for the choice of cases and controls  *Cross-sectional study*—Give the eligibility criteria, and the sources and methods of selection of participants | 5 | Inclusion: “Patients were eligible if they had at least one documented postoperative assessment with a measured RERR.” Exclusion: “Patients with bilateral lymphedema, bilateral interventions, with additional liposuction and/or without written informed consent were excluded.” The meaning of minimum follow-up is defined: “‘minimum follow-up’ refers to the availability of at least one postoperative measurement rather than completion of the full 24-month schedule.” |
|  |  | (*b*) *Cohort study*—For matched studies, give matching criteria and number of exposed and unexposed  *Case-control study*—For matched studies, give matching criteria and the number of controls per case | - | Not applicable. This is an unmatched cohort; neither propensity-score matching nor other matching was performed. The resulting confounding by indication is addressed in the Limitations (Section 4.3). |
| Variables | 7 | Clearly define all outcomes, exposures, predictors, potential confounders, and effect modifiers. Give diagnostic criteria, if applicable | 5-7 | Primary outcome: relative excess reduction rate (RERR), defined in Section 2.3 with its sign convention in Section 2.4 (“a negative RERR reflects a reduction of the excess circumference relative to baseline (i.e., clinical improvement)”). Exposures/predictors: surgical technique (LVA vs. VLNT), extremity (upper vs. lower) and time. Potential confounders (age, BMI, lymphedema aetiology and stage) are reported in Tables 1–3 and 6; the differing postoperative compression protocols are described in Section 2.2 and discussed as a time-varying co-intervention.  Secondary outcome: postoperative complications graded by the Clavien-Dindo classification. |
| Data sources/ measurement | 8* | For each variable of interest, give sources of data and details of methods of assessment (measurement). Describe comparability of assessment methods if there is more than one group | 5,7 | Section 2.1: standardized circumference measurement at predefined landmarks “at 10 cm intervals extending proximally up to 50-60 cm from the wrist or ankle”, performed “by specially trained plastic surgeons following the standardized protocols” at all scheduled visits; clinical variables and complications extracted from medical records. Section 2.4 justifies the metric and states which alternatives were unavailable: “Perometry, bioimpedance spectroscopy, volumetric imaging and water-displacement volumetry were not routinely available.” The same protocol was applied in both groups and both extremities. |
| Bias | 9 | Describe any efforts to address potential sources of bias | 5-7, 15-16 | Measurement bias: standardized landmarks, trained assessors, and the unaffected contralateral limb as internal reference for every patient. Selection bias: procedure allocation was clinically indicated, therefore the analysis is framed as a two-group longitudinal comparison “not intended to establish superiority of one technique over the other” (Section 2.1) and confounding by indication is stated explicitly in Section 4.3. Co-intervention bias: the differing compression protocols are named as “a time-varying co-intervention represents a potential source of bias”. |
| Study size | 10 | Explain how the study size was arrived at | 5 | No a priori sample size calculation was performed. The study size corresponds to all patients in the database who met the eligibility criteria within the predefined period (January 2016 to December 2023), resulting in 265 patients. |

Continued on next page

| Quantitative variables | 11 | Explain how quantitative variables were handled in the analyses. If applicable, describe which groupings were chosen and why | 7-8 | Section 2.4: “For each limb, the circumferences measured at the predefined landmarks were aggregated as an arithmetic mean (⌀), and the RERR was calculated from the mean-circumference difference between the affected and unaffected limb, normalized to the preoperative excess.” RERR was analysed as a continuous variable. Time was categorized at the scheduled follow-up visits: “Time was modeled as a categorical variable (3, 6, 12, 24 months; 3 months as reference).” |
| --- | --- | --- | --- | --- |
| Statistical methods | 12 | (*a*) Describe all statistical methods, including those used to control for confounding | 7-8 | Descriptive statistics; Fisher’s exact test for categorical and Wilcoxon rank-sum test for continuous variables. “To assess changes in RERR over time and between intervention groups, we used linear mixed-effects models with a random intercept for each patient … fixed effects included intervention (LVA/VLNT) and extremity (upper/lower) together with their two- and three-way interactions with time.” Estimated marginal means with 95% CI were derived from the fitted models; full model coefficients are given in Supplementary Tables S1–S3. No additional multivariable confounder adjustment was performed; this is stated as a limitation (Section 4.3). |
|  |  | (*b*) Describe any methods used to examine subgroups and interactions | 7-8, 10-11 | Subgroups (extremity, procedure) were examined through the interaction terms of the mixed models and through separate models for LVA (Section 3.2, Supplementary Table S2) and VLNT (Section 3.3, Supplementary Table S3). p-values for time, group and interaction terms are reported. |
|  |  | (*c*) Explain how missing data were addressed | 7-8,10 | No imputation was performed. The linear mixed-effects models use all available repeated measurements per patient, so that patients with incomplete follow-up contribute to the timepoints at which a measurement was obtained. The number of patients with an available RERR at each timepoint is reported in Table 4. |
|  |  | (*d*) *Cohort study*—If applicable, explain how loss to follow-up was addressed  *Case-control study*—If applicable, explain how matching of cases and controls was addressed  *Cross-sectional study*—If applicable, describe analytical methods taking account of sampling strategy | 10, 15-16 | Loss to follow-up is quantified in Table 4 (patients assessed at each timepoint by procedure and extremity) and discussed in Section 4.3: “This attrition was substantial and likely nonrandom … Because dropout was probably related to outcome, the long-term trajectory estimates, particularly for LVA, may be biased, and long-term comparisons between procedures should be interpreted with caution.” |
|  |  | (*e*) Describe any sensitivity analyses | 15-16 | No sensitivity analyses were performed. This is stated explicitly in Section 4.3: “There was no formal comparison of baseline characteristics between patients with and without long-term follow-up or a sensitivity analysis for informative dropout, which represents further limitations …” |
| Results | | | | |
| Participants | 13* | (a) Report numbers of individuals at each stage of study—eg numbers potentially eligible, examined for eligibility, confirmed eligible, included in the study, completing follow-up, and analysed | 9-11 | Section 3: “A total of 265 patients were enrolled in this study …”, of whom 151 (57.0%) had lower and 114 (43.0%) upper extremity lymphedema; 166 (62.6%) underwent LVA and 99 (37.4%) VLNT. The number of patients assessed at each follow-up, stratified by procedure and extremity, is reported in Table 4. |
|  |  | (b) Give reasons for non-participation at each stage | 5, 10, 15-16 | Reasons for exclusion are listed in Section 2.1 (bilateral lymphedema, bilateral intervention, additional liposuction, missing written consent). Reasons for incomplete follow-up are discussed in Section 4.3 (probable outcome-related dropout after early improvement; additional donor-site surveillance after VLNT increasing attendance). |
|  |  | (c) Consider use of a flow diagram | 10 | Instead of a flow diagram, Table 4 reports the number of patients assessed and the proportion with RERR improvement at each follow-up, separately for LVA and VLNT and for both extremities. |
| Descriptive data | 14* | (a) Give characteristics of study participants (eg demographic, clinical, social) and information on exposures and potential confounders | 9 | Baseline characteristics (age, BMI, sex, aetiology, lymphedema stage, surgical technique) are shown in Table 1, procedure-specific characteristics in Tables 2, 3 and 6, and summarized in the first paragraph of the Results. |
|  |  | (b) Indicate number of participants with missing data for each variable of interest | 9-10 | Missing or unavailable baseline information is reported as “N/A” rows in Tables 1, 2 and 6. The number of patients with an available RERR measurement per timepoint is reported in Table 4. |
|  |  | (c) *Cohort study*—Summarise follow-up time (eg, average and total amount) | 5, 9-11 | Follow-up was scheduled at 3, 6, 12 and 24 months (Section 2.1); the maximum follow-up analysed was 24 months. Table 4 summarizes the number of patients contributing data at each of these timepoints. |
| Outcome data | 15* | *Cohort study*—Report numbers of outcome events or summary measures over time | 10-12 | RERR estimates with 95% CI over time are reported in Table 5 (LVA) and Table 7 (VLNT) and displayed in Figures 1–3. Proportions of patients with RERR < 0 per timepoint are given in Table 4; at 24 months “70 of 89 patients (78.7%) had a RERR < 0”. Complications are reported in Section 3.4 and Table 8 (11 events, 4.2%). |
|  |  | *Case-control study—*Report numbers in each exposure category, or summary measures of exposure |  |  |
|  |  | *Cross-sectional study—*Report numbers of outcome events or summary measures |  |  |
| Main results | 16 | (*a*) Give unadjusted estimates and, if applicable, confounder-adjusted estimates and their precision (eg, 95% confidence interval). Make clear which confounders were adjusted for and why they were included | 10-12 | Estimated marginal means with 95% CI from the mixed-effects models are reported in Tables 5 and 7; complete model coefficients with 95% CI and p-values are provided in Supplementary Tables S1–S3. The models include time, intervention, extremity and their interactions; no further confounders were adjusted for, and the resulting confounding by indication is stated in Section 4.3. Statements of statistical significance are restricted to those groups and timepoints whose confidence intervals exclude zero. |
|  |  | (*b*) Report category boundaries when continuous variables were categorized | 7-8 | Time was categorized according to the scheduled follow-up visits (3, 6, 12 and 24 months, with 3 months as reference). No other continuous variable was categorized. |
|  |  | (*c*) If relevant, consider translating estimates of relative risk into absolute risk for a meaningful time period | - | Not applicable. No relative risk estimates were calculated; the outcome is a continuous relative measure (RERR). |

Continued on next page

| Other analyses | 17 | Report other analyses done—eg analyses of subgroups and interactions, and sensitivity analyses | 10-12 | Subgroup analyses by extremity and by procedure are reported in Sections 3.1–3.3 with the corresponding interaction terms in Supplementary Tables S1–S3. Table 4 additionally compares the proportion of improved patients between LVA and VLNT at each timepoint (Fisher’s exact test). No sensitivity analyses were performed (see item 12e). |
| --- | --- | --- | --- | --- |
| Discussion | | | | |
| Key results | 18 | Summarise key results with reference to study objectives | 13 | First paragraph of the Discussion: “Our findings demonstrate that both LVA and VLNT are effective in treating lymphedema with low complication rates. However, their temporal improvement profiles differ substantially between the upper and lower extremities.” Each subsection of the Discussion opens with the corresponding key result. |
| Limitations | 19 | Discuss limitations of the study, taking into account sources of potential bias or imprecision. Discuss both direction and magnitude of any potential bias | 15-16 | Section 4.3 states the observational, non-randomized design and confounding by indication (“Because procedure allocation was based on clinical indication, confounding by indication cannot be excluded and precludes a direct head-to-head comparison of the two techniques.”), the substantial and probably outcome-related attrition together with its likely direction of bias, the differing compression protocols as a time-varying co-intervention, the circumference-derived rather than volumetric outcome, and the absence of PROMs and of sensitivity analyses. |
| Interpretation | 20 | Give a cautious overall interpretation of results considering objectives, limitations, multiplicity of analyses, results from similar studies, and other relevant evidence | 16 | *Section 4.3 concludes: “Consequently, these factors mean that the findings should be interpreted as a two-group longitudinal analysis rather than as evidence of the superiority of one technique over the other.” The Conclusion is phrased accordingly (“Temporal clinical response to LVA or VLNT were observed to differ …”) and calls for further validation in future trials.* |
| Generalisability | 21 | Discuss the generalisability (external validity) of the study results | 15-16 | Section 4.3: the incomplete long-term follow-up “represents further limitations and reduces the generalizability of the long-term findings”; in addition, the predominance of stage II lymphedema limits conclusions on stage-specific efficacy (Section 4.1). |
| Other information | |  | | |
| Funding | 22 | Give the source of funding and the role of the funders for the present study and, if applicable, for the original study on which the present article is based | 20 | Declarations: “This study was not funded.” |

*Give information separately for cases and controls in case-control studies and, if applicable, for exposed and unexposed groups in cohort and cross-sectional studies.

**Note:** An Explanation and Elaboration article discusses each checklist item and gives methodological background and published examples of transparent reporting. The STROBE checklist is best used in conjunction with this article (freely available on the Web sites of PLoS Medicine at http://www.plosmedicine.org/, Annals of Internal Medicine at http://www.annals.org/, and Epidemiology at http://www.epidem.com/). Information on the STROBE Initiative is available at www.strobe-statement.org.
