# Supplementary material for: Vascularized lymph node transfer and lymphovenous anastomosis: temporal microsurgical outcomes in upper versus lower extremity lymphedema
Source: JPRAS Open. 2026 Oct 20;53:128–38. doi: 10.1016/j.jpra.2026.10.011 (PMC13761365; doi:10.1016/j.jpra.2026.10.011)
Supplement: Supplementary file 2 [file mmc2.docx]

**Supplementary Table S1. Linear mixed-effects regression of relative excess reduction rates by procedure and extremity over time**

| **Coefficients** | **Estimate** | **95% CI** | **p-value** |
| --- | --- | --- | --- |
| (Intercept) | −126.7 | −215.5 to −38.0 |  |
| **Time** |  |  | **0.333** |
| 6 months | 72.25 | −4.07 to 148.7 |  |
| 12 months | 77.06 | −5.37 to 159.4 |  |
| 24 months | 78.05 | −4.25 to 160.2 |  |
| **VLNT** | 47.07 | −49.93 to 144.4 | **0.498** |
| **Leg** | 87.16 | −26.26 to 200.3 | **0.141** |
| **Interaction Time:VLNT** |  |  | **0.229** |
| 6 months:VLNT | −46.60 | −127.3 to 33.78 |  |
| 12 months:VLNT | −39.87 | −126.6 to 46.91 |  |
| 24 months:VLNT | −31.45 | −119.8 to 56.91 |  |
| **Interaction Time:Leg** |  |  | **0.022** |
| 6 months:Leg | −27.28 | −127.1 to 72.40 |  |
| 12 months:Leg | −56.59 | −162.4 to 49.66 |  |
| 24 months:Leg | −107.2 | −221.6 to 9.92 |  |
| **VLNT:Leg** | 25.12 | −126.4 to 177.8 | **0.970** |
| **Interaction Time:VLNT:Leg** |  |  | **0.912** |
| 6 months:VLNT:Leg | −43.37 | −172.9 to 85.82 |  |
| 12 months:VLNT:Leg | −32.91 | −168.2 to 101.3 |  |
| 24 months:VLNT:Leg | −16.10 | −167.9 to 132.3 |  |

*Coefficients are from a linear mixed-effects model of the relative excess reduction rate (RERR, %) with a random intercept per patient. Time was modelled as a categorical variable with the 3-month visit as the reference level (included in the intercept). More negative RERR values indicate greater reduction of excess volume. Bolded rows give the p-value for the corresponding fixed effect or interaction term. CI Confidence interval; LVA Lymphovenous anastomosis; VLNT Vascularized lymph node transfer.*

**Supplementary Table S2. Linear mixed-effects regression of relative excess reduction rates by extremity over time for LVA**

| **Coefficients** | **Estimate** | **95% CI** | **p-value** |
| --- | --- | --- | --- |
| (Intercept) | −127.2 | −230.0 to −24.59 |  |
| **Time** |  |  | **0.181** |
| 6 months | 73.28 | −4.58 to 151.2 |  |
| 12 months | 76.15 | −7.89 to 160.1 |  |
| 24 months | 76.89 | −6.93 to 160.6 |  |
| **Leg** | 86.03 | −46.13 to 217.9 | **0.393** |
| **Interaction Time:Leg** |  |  | **0.449** |
| 6 months:Leg | −28.33 | −131.3 to 74.55 |  |
| 12 months:Leg | −53.74 | −162.7 to 55.53 |  |
| 24 months:Leg | −88.88 | −207.0 to 30.88 |  |

*Coefficients are from a linear mixed-effects model of the relative excess reduction rate (RERR, %) with a random intercept per patient. Time was modelled as a categorical variable with the 3-month visit as the reference level (included in the intercept). More negative RERR values indicate greater reduction of excess volume. Bolded rows give the p-value for the corresponding fixed effect or interaction term. CI Confidence interval; LVA Lymphovenous anastomosis.*

**Supplementary Table S3. Linear mixed-effects regression of relative excess reduction rates by extremity over time for VLNT**

| **Coefficients** | **Estimate** | **95% CI** | **p-value** |
| --- | --- | --- | --- |
| (Intercept) | −83.83 | −107.7 to −59.66 |  |
| **Time** |  |  | **0.967** |
| 6 months | 32.71 | 8.01 to 57.58 |  |
| 12 months | 40.00 | 13.70 to 66.37 |  |
| 24 months | 51.16 | 19.75 to 82.67 |  |
| **Leg** | 96.54 | 25.08 to 168.7 | **0.045** |
| **Interaction Time:Leg** |  |  | **0.178** |
| 6 months:Leg | −55.26 | −130.1 to 19.51 |  |
| 12 months:Leg | −64.89 | −141.6 to 11.93 |  |
| 24 months:Leg | −101.8 | −190.4 to −13.18 |  |

*Coefficients are from a linear mixed-effects model of the relative excess reduction rate (RERR, %) with a random intercept per patient. Time was modelled as a categorical variable with the 3-month visit as the reference level (included in the intercept). More negative RERR values indicate greater reduction of excess circumference. Bolded rows give the p-value for the corresponding fixed effect or interaction term. CI Confidence interval; VLNT Vascularized lymph node transfer.*
